# Two-centre comparative experimental study of biparametric MRI at 3.0 T with and without endorectal coil using kiwifruit (*Actinidia deliciosa*) as a phantom for human prostate

**DOI:** 10.1186/s41747-019-0111-8

**Published:** 2019-08-14

**Authors:** Sophie Murer, Juergen Scheidler, Ulrike L. Mueller-Lisse, Marissa Helling, Michael Scherr, Ullrich G. Mueller-Lisse

**Affiliations:** 1 Department of Radiology, Faculty of Medicine, University of Munich (“Ludwig-Maximilians-Universität”, LMU), Ziemssenstrasse 1, 80336 Muenchen, Germany; 2 Department of Radiology, Radiology Centre Munich (RZM), Muenchen, Germany; 3 Department of Urology, Faculty of Medicine, University of Munich (Ludwig-Maximilians-Universität, LMU), Munich, Germany; 4 Department of Urology, Interdisciplinary Oncology Centre Munich (IOZ), Munich, Germany; 5 Department of Radiology, BG Unfallklinik Murnau, Murnau am Staffelsee, Germany

**Keywords:** *Actinidia deliciosa*, Diffusion-weighted imaging, Magnetic resonance imaging, Phantoms (imaging), Prostate

## Abstract

**Background:**

Application of an endorectal coil (ERC) for 3.0-T prostate magnetic resonance imaging (MRI) is contentious. We hypothesised that a multicoil phased-array protocol provides T2-weighted images (T2WI) and diffusion-weighted images (DWI) with reduced field-of-view (DWI_reduced_) and monoexponential apparent diffusion coefficient (ADC) maps that are technically equivalent with ERC or without ERC (noERC).

**Methods:**

Axial T2WI (repetition time [TR] 7500 ms, echo time [TE] 98–101 ms) and DWI_reduced_ (field-of-view 149–179 × 71–73 mm^2^, TR/TE 4500–5500/61–74 ms, *b* values, 50/800 s/mm^2^) ERC and noERC images were obtained on identical clinical 3.0-T scanners at two centres and compared for signal-to-noise ratio (SNR) in anterior and posterior outer pericarp (OP) and peripheral placenta (PP) in five green Hayward kiwifruit (*Actinidia delicios*a, European Union regulation 543/2011 class 2). Corroboration in 21 patients with benign prostate hyperplasia (negative biopsy, prostate imaging reporting and data system version 2 ≤ 2) involved identical MRI protocols: 10 at site 1, noERC, and 11 at site 2, with ERC. Two-tailed Student’s *t* test was used.

**Results:**

With few exceptions, signal-to-noise ratio (SNR) was similar in kiwifruits and prostates for ERC and noERC. In T2WI, SNR was higher posteriorly in noERC MRI for peripheral zone (PZ) (*p* < 0.001). In DWI_reduced_, SNR was higher posteriorly in ERC-OP (*p* = 0.013) and ERC-PZ (*p* = 0.026) for *b* = 50 s/mm^2^; noERC-OP (*p* = 0.044) and ERC-PZ (*p* = 0.001) for *b* = 800 s/mm^2^; and ERC-OP (*p* = 0.001), noERC-OP (*p* = 0.001), and ERC-PZ (*p* = 0.001) for ADC, respectively. Volumes of kiwifruits and prostates were similar (89.2 ± 11.2 versus 90.8 ± 48.5 cm^3^, *p* = 0.638–0.920).

**Conclusions:**

Findings imply that multicoil phased-array 3.0-T prostate MRI with T2WI and DWI_reduced_ with ADC maps provides equivalent results with and without ERC.

## Key points

- Physical properties of kiwifruits are standardised by the European Union regulation 543/2011
- With endorectal coil, signal-to-noise ratio (SNR) was similar for kiwifruits and prostates in T2-weighted and diffusion-weighted 3.0-T magnetic resonance imaging (MRI)
- Without endorectal coil, SNR was similar for kiwifruits and prostates in T2-weighted and diffusion-weighted 3.0-T MRI
- Kiwifruits may substitute for prostates in testing T2-weighted and diffusion-weighted 3.0-T MRI

## Background

Multiparametric magnetic resonance imaging of the prostate (MRI) has evolved to be a powerful clinical tool both in the detection and staging of prostate cancer and in guiding prostate biopsy. It comprises T2-weighted imaging (T2WI), diffusion-weighted imaging (DWI), dynamic contrast-enhanced imaging, and magnetic resonance spectroscopic imaging [1–3]. Among these, T2WI and DWI with apparent diffusion coefficient (ADC) maps are currently perceived to be most decisive for diagnosis [4–7].

Nonetheless, variability in the technical approach to multiparametric MRI is common despite recent attempts in standardisation [1–4, 8]. The application of an endorectal coil (ERC) for multiparametric MRI at 3.0 T is one contentious issue. An ERC may improve test quality parameters [9–11]. However, the potential advantage comes at the expense of increased cost, workflow challenges, and patient discomfort [6]. Also, the assumption that signal-to-noise ratio (SNR) would improve with ERC application, due to closer proximity of the receiving coil to the prostate, has been challenged in a recent clinical study [6]. In addition, ERCs induce significant inhomogeneity in signal intensity [12, 13]. Correction algorithms applied within the scanner may lead to noise amplification and noise level variation [12]. Different external compensation methods have been developed and tested in mathematical simulations, custom-made phantoms, and clinical data [12, 13]. However, although such technical compensation methods may be underway, the question remains if in the clinical setting, with commercially available MRI equipment, there is an SNR improvement using ERC for prostate 3.0-T imaging.

Lately, commercially available kiwifruits (*Actinidia deliciosa*) have been introduced as a phantom model to substitute for human prostates in dedicated MR examinations including T2WI, DWI with ADC maps, and magnetic resonance spectroscopy [14]. Unlike human prostates, which are known for biological variability [15–17], kiwifruits traded in the European Union are regulated to limit biological differences [18]. Thus, experiments comparing different MRI techniques involving kiwifruits instead of human prostates should emphasise technical differences and limit effect modification by biological variability. To answer the question if ERC application improves SNR in prostate imaging at 3.0 T, we therefore examined kiwifruits in a biparametric MRI protocol for prostate imaging that included T2WI and DWI with a reduced field-of-view (DWI_reduced_) and ADC maps.

We hypothesised that prostate MRI provides technically equivalent results using ERC or not using ERC (noERC) for a biparametric protocol including T2WI and DWI_reduced_ with monoexponential ADC maps. The hypothesis was tested using kiwifruits as phantoms at two centres (site 1 and site 2) which operated identical 3.0-T scanners albeit with small variations in the protocols for T2WI and DWI. Site 1 routinely examined patients without ERC, while site 2 routinely applied ERCs. Findings in kiwifruits were retrospectively compared with measurements obtained in patients with benign prostate hyperplasia from each of the two sites.

## Methods

### Study design, setting, and participants

In a two-centre (sites 1 and 2) combined prospective-experimental and retrospective-clinical study performed from August 2015 to February 2018, five fresh, firm green kiwifruits (Hayward varietal, *Actinidia deliciosa*, European Union regulation 543/2011 class 2, produced in Italy) were consecutively subjected to axial T2WI and DWI_reduced_ sequences at 3.0 T, with and without ERC, at ambient room temperature, pressure, and humidity. T2WI and DWI_reduced_ were also obtained for another kiwifruit, using an ERC filled with air, at both centres. The same kiwifruit was examined with the same ERC subsequently filled with perfluorocarbon (PFC) in site 2.

Retrospective comparison of T2WI and DWI_reduced_ in kiwifruits and in human prostates of selected patients with BPH was approved by the institutional review board and individual consent was waived. All patients, aged 62 ± 8 years (mean ± standard deviation [SD]), had elevated prostate-specific antigen serum levels (6.3 ± 2.4 ng/mL, mean ± SD) and previously negative prostate biopsy results and were referred to prostate MRI to corroborate biopsy findings and rule out undetected prostate cancer lesions. All patients had prostate imaging reporting and data system version 2 (PI-RADS-v2) scores of “1” or “2”at MRI. Ten patients had been examined without ERC in site 1 and eleven patients with ERC in site 2, applying the same scanners and protocols as for kiwifruits.

### Variables

Signal-to-noise ratio (SNR) was calculated for tissues of interest in both kiwifruits and human prostates by dividing the signal intensity value deriving from magnitude image data provided by the standard image reconstruction algorithm (SI) by its first SD as a combined measure of noise and tissue homogeneity within the same region of interest (ROI). Tissues of interest included the outer pericarp (OP) and peripheral placenta (PP) of kiwifruits and the peripheral zone (PZ) and transitional zone (TZ) of human prostates (Fig. 2). ERC application and differences between sites in MRI-protocols for T2WI and DWI_reduced_ were considered as effect modifiers. Potential confounders were the respective sizes of kiwifruits and prostates, i.e. their anterior-posterior, lateral, and cranio-caudal diameters as measured in T2WI, and their volume as determined by the ellipsoid formula (volume = anterior-posterior diameter × lateral diameter × cranio-caudal diameter × π/6), the respective anterior-posterior and lateral diameters of the kiwifruit phantom at the level of the largest anterior-posterior and lateral diameters of kiwifruit and of the human pelvis at the level of the largest anterior-posterior and lateral diameters of prostates as measured in localiser images, and the respective distances of the posterior surface of kiwifruits and prostates from the table top of the scanner as measured on localiser images.

### Kiwifruit phantom

**Fig. 1:**
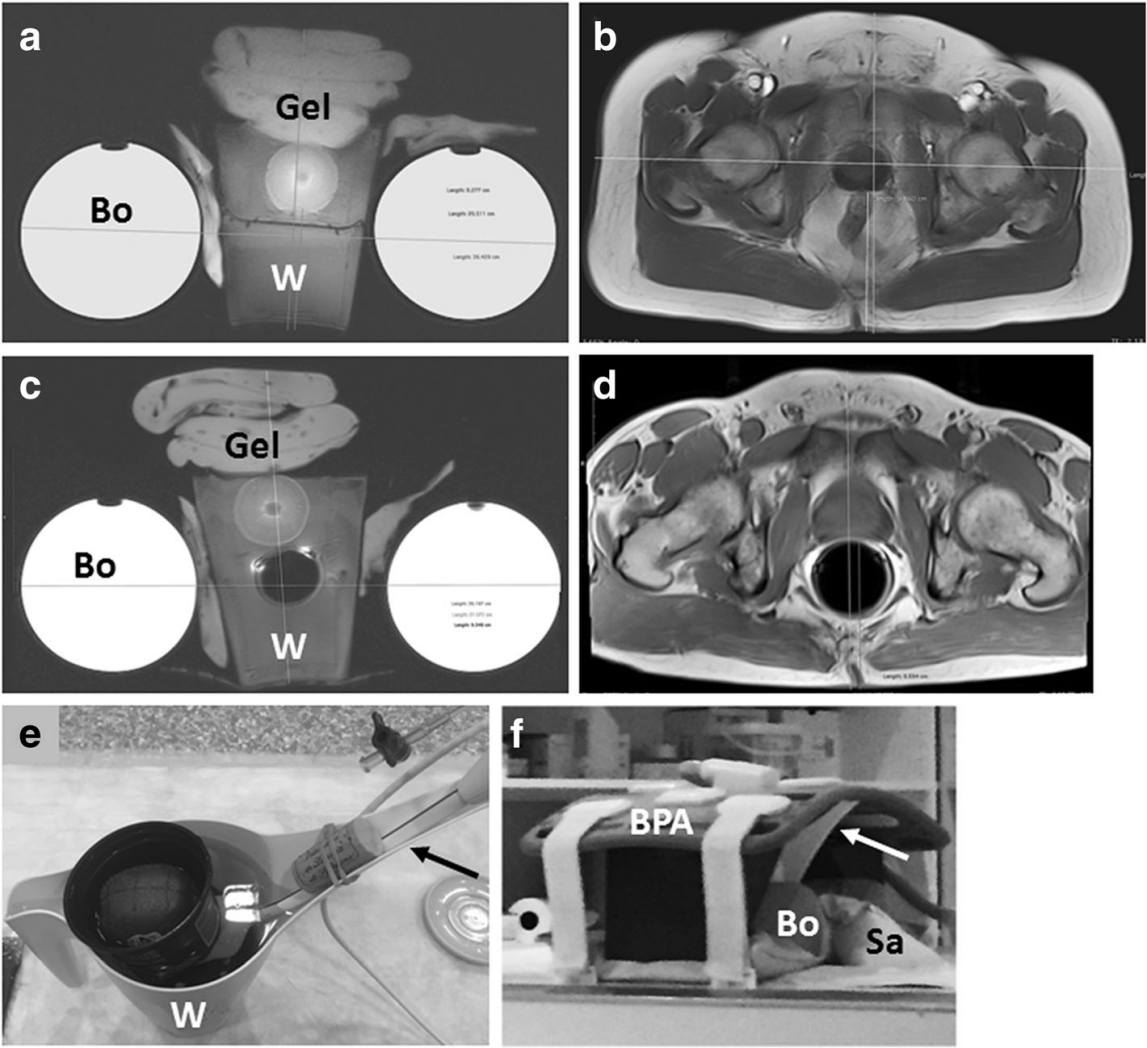
Setup of kiwifruit phantom and comparison with a male human pelvis. Respective axial MR-images of kiwifruit phantom (**a**, **c**) and human pelvis (**b**, **d**) examined with bi-parametric magnetic resonance imaging at 3.0 T without (**a**, **b**) and with an endo-rectal coil (**c**, **d**) included in a multichannel phased-array coil system show similar coil-load; “Bo” indicates copper-sulphate solution bottle, “Gel” indicates gel compress-packs, and “W” indicates open-spout plastic watering-can. Photographs (**e**, **f**) show a watering can (W) with endo-rectal coil handle fixed in the spout (arrow) with a wine-cork and rubber-band, and set-up complete with supporting sandbags (Sa) and abdominal phased-array surface coil (BPA)

Setup of the kiwifruit phantom followed previous descriptions [14]. In short, kiwifruits were fixed with several 12 × 29-cm gel compress packs (e.g., Koolpak, Poole, Dorset, England, UK) in the upper one of two plastic nursery pots (e.g., VTG 9, TEKU Poeppelmann, Lohne, Germany), such that either the stalk side or the blossom side pointed toward the scanner (Fig. 1a, c, e). Nursery pots were placed one on top of the other, separated either by a plastic plate (diameter, approximately 10 cm) for MRI without ERC (Fig. 1a), or by the ERC balloon (inflated to 60 mL, Fig. 1c, e), and submerged in tap water inside a 1-L open-spout plastic watering can (e.g. “Oase”, Elho, Tilburg, The Netherlands), with the ERC handle fixed in the spout with a wine cork and rubber band (Fig. 1e). The watering can was stabilised by means of gel compress packs, sandbags, copper-sulphate solution bottles, and foamed rubber supports, such that the setup resembled an adult human pelvis in anterior-posterior and lateral diameters and coil-load for pelvic imaging (Fig. 1a, d). A disposable cellulose kidney dish was placed under the handle of the watering can and a disposable incontinence draw sheet underneath the phantom. The abdominal phased-array surface coil was placed on top, such that it pressed down onto the compress packs, and fixed to the scanner table per vendor’s recommendation for abdominal and pelvic MRI (Fig. 1f).

### MRI

At both sites, kiwifruits and patients with benign prostate hyperplasia were examined on same-model commercially available clinical MRI 3.0-T systems (Magnetom Skyra, software version Syngo MR E11, Siemens Medical Solutions, Erlangen, Germany), with the body coil for transmission and a phased-array coil system for signal reception that included elements of a 32-channel spine coil and an 18-channel body surface coil. A commercially available ERC (3.0-T Prostate eCoil, Medrad Europe BV, Maastricht, The Netherlands) was included as a single-use item into the phased-array coil system for MRI with ERC. Sequences and parameters evaluated in this study are displayed in Table 1.

**Table 1:** Magnetic resonance sequences and parameters for axial imaging in kiwifruits and human prostates at two different institutions (site 1, site 2)

Parameter	T2WI site 1	T2WI site 2	DWI_reduced_ site 1	DWI_reduced_ site 2
Field of view (mm^2^)	180 × 180	160 × 160	149 × 73	179 × 71
Imaging matrix	320 × 320	320 × 320	90 × 44	140 × 42
Slice thickness (mm)	3.0	3.0	3.5	3.5
Slice spacing (mm)	3.0	3.3	3.5	3.5
Voxel size (mm^3^)	0.56 × 0.56 × 3.00	0.50 × 0.50 × 3.00	1.66 × 1.66 × 3.50	1.28 × 1.69 × 3.50
No. of slices	26	30	20	22
PAT mode	GRAPPA	GRAPPA	n/a	n/a
Acceleration factor PE	2	2	n/a	n/a
Echo time (ms)	101	98	74	74
Echo train length	25	17	38	42
Time to repeat (ms)	7500	7400	4500	4500
Bandwidth (Hz/px)	200	180	1500	1190
*b* value (s/mm^2^)	n/a	n/a	50, 400, 800	50, 800
Number of excitations	3	2	2, 5, 12 (per *b* value)	4, 16 (per *b* value)
Acquisition time (min:s)	04:22	04:38	04:32	04:44
Flip angle	160°	120°	90°	90°
Percent sampling	100	100	100	75
Percent phase field of view	100	100	48.9	40

*ADC* apparent diffusion coefficient, *DWI*_*reduced*_ echo-planar diffusion-weighted imaging with reduced field of view and selective excitation, *GRAPPA* generalised autocalibrating partially parallel acquisition, *n/a* not applicable, *PAT* parallel acquisition technique, *T2WI* T2-weighted turbo-spin-echo imaging

### Measurements

Examinations were anonymised, stored in and retrieved for evaluation from the picture archiving and communication system of site 1 (PACS, syngo studio VB36C, syngo imaging VB36C,

Siemens Healthcare Systems, Erlangen, Germany), and displayed on 5-K monitors licensed for clinical image interpretation. Quantitative variables were measured by two researchers working in consensus on axial images obtained at the largest transverse diameter and 2 cm toward the blossom and toward the stalk of kiwifruits, respectively, and at the widest lateral diameter, i.e. the most cranial level of the middle, the first level of the apex, i.e. the first level caudal (inferior) to the verumontanum, and at the base of prostate, cranial (superior) to the widest lateral diameter, respectively [19].

**Fig. 2:**
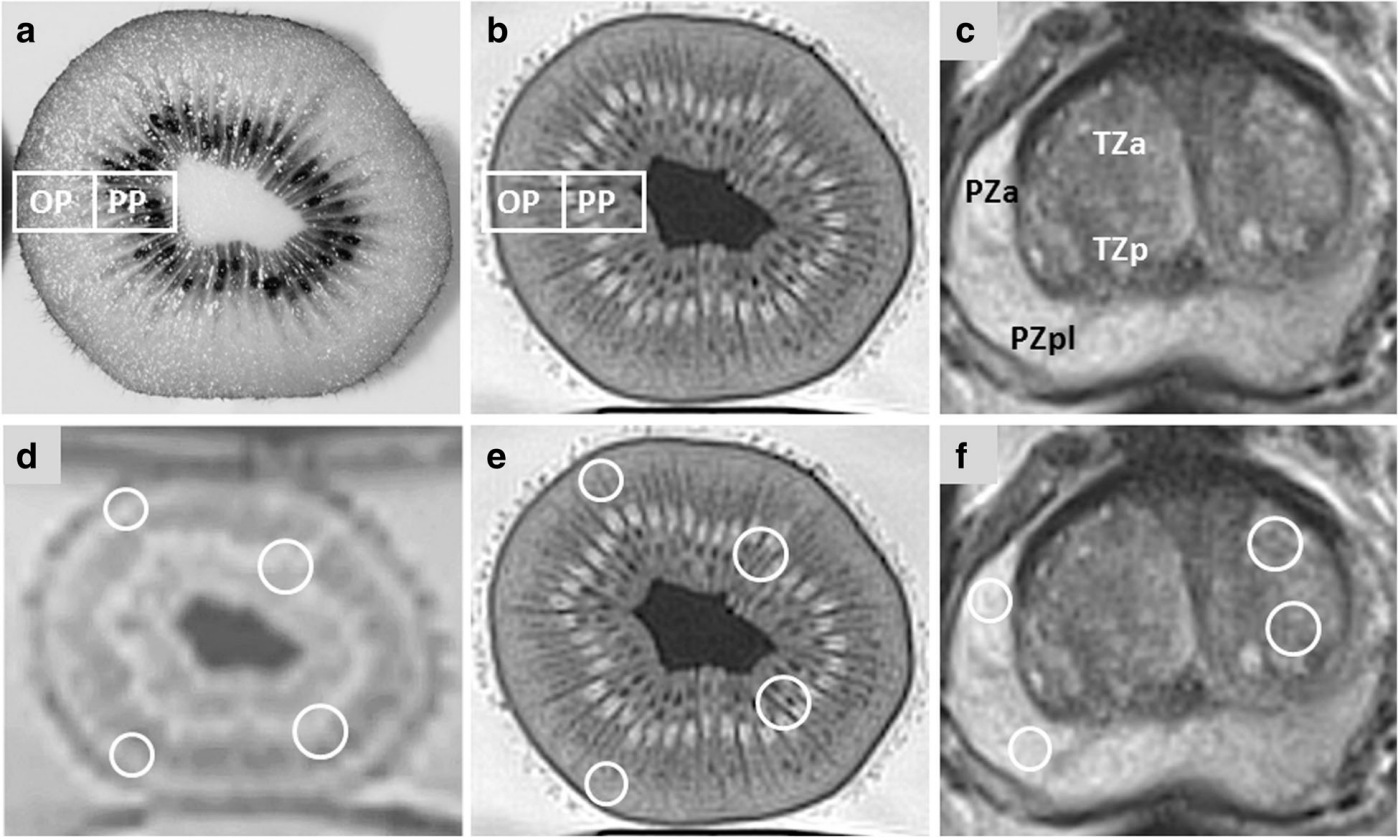
Axial images of a green Hayward kiwifruit, *Actinidia deliciosa* (**a** digital photograph of fresh cut section; **b** corresponding T2-weighted image [T2WI]; OP, outer pericarp; PP, peripheral placenta) and a human prostate (c T2WI: PZa, anterior region of peripheral zone; PZpl, posterior region of peripheral zone; TZa, anterior region of transitional zone; TZp, posterior region of transitional zone). Respective placement of elliptical regions of interest (ROIs) comprising at least ten adjacent voxels is demonstrated in axial images of a kiwifruit (**d** diffusion-weighted image, *b* value 800 s/mm^2^; **e** T2WI) and human prostate (**f** T2WI)

Quantitative measurements derived from elliptical ROIs comprising at least 10 adjacent voxels (Fig. 2). Using tools available from the picture archiving and communication system, SI (arbitrary units) was recorded as mean ± SD in each, OP, PP, PZ, and TZ, in T2WI, and DWI_reduced_, with low (*b* = 50 s/mm^2^) and high (*b* = 800 s/mm^2^) *b* values and mono-exponential ADC maps, respectively. In each axial level, measurements in kiwifruits were obtained in anterior and posterior positions in each OP and PP. Measurements in human prostates were gained in the left and right anterior and posterior (lateral) anatomical regions of TZ and PZ, respectively, as per the 39-region scheme provided by PI-RADS-v2 [4].

### Bias

All examinations were performed on the same scanner model, applying a similar sequence protocol in both kiwifruits and human prostates, with small differences between sites (Table 1). Kiwifruits were examined both with and without ERC at both sites, allowing for intra-individual comparison. Measurements in human prostates were limited to patients with benign prostate hyperplasia, with PI-RADS-v2 scores of 1 or 2 and prostate biopsy results without evidence of prostate cancer or severe prostatitis. However, patients at site 1 were examined without ERC only, while patients at site 2 were examined with ERC only. To avoid bias by differences in patients and differences in technical parameters other than ERC application, statistical evaluation of SNR in patients was limited to intra-individual anterior-posterior comparison.

### Study size

Although very little inter-individual variability of physical properties was expected in kiwifruits, due to pertinent European Union regulations [18], we included several kiwifruits to reflect the remaining variation. From the prostate MRI databases of both sites, we selected examinations of 21 patients with benign prostate hyperplasia, including ten examined without ERC as per prostate protocol of site 1, and eleven with ERC as per prostate protocol of site 2, to reflect variance in prostate diameters and volume.

### Quantitative variables and statistical methods

The SNR (in arbitrary units) was tabulated as mean ± SD for respective anterior and posterior regions within OP and PP of kiwifruits, and PZ and TZ of human prostates, respectively. Two-sided Student’s *t* test for paired samples (intra-individual comparisons of SNR, distances/diameters, and volumes, Tables 2, 3, 4, 5) or unpaired samples (inter-individual comparisons of distances/diameters and volumes, Table 5) [20] were performed in Microsoft Excel 2003 (Microsoft Corporation, Redmond, WA, USA) To account for multiple testing involving the same data, statistical significance was assumed for *p* < 0.005. As a consequence, test results of 0.005 < *p* < 0.050 were reported as being not significantly different.

**Table 2:** Signal-to-noise ratios in kiwifruits examined with and without an endorectal coil at site 1 (twelve corresponding regions of interest in anterior and posterior locations) and at site 2 (three corresponding regions of interest in anterior and posterior locations)

Kiwifruits, outer pericarp, site 1	ERC, *n* = 12 × 2 ROIs			noERC, *n* = 12 × 2 ROIs			ERC versus noERC	
	SNR, anterior (mean ± SD)	SNR, posterior (mean ± SD)	*p* value	SNR, anterior (mean ± SD)	SNR, posterior (mean ± SD	*p* value	SNR, anterior*,p* value	SNR, posterior*,p* value
T2WI	14.2 ± 2.4	13.9 ± 3.7	*0.742*	13.3 ± 2.4	13.1 ± 2.7	*0.763*	*0.428*	*0.546*
DWI_reduced_ *b* = 50	4.8 ± 2.4	7.8 ± 2.7	0.013	7.4 ± 4.0	8.2 ± 2.4	*0.541*	0.005	*0.688*
DWI_reduced_ *b* = 800	7.6 ± 7.1	7.3 ± 2.8	*0.894*	7.0 ± 3.2	10.7 ± 4.7	0.044	*0.736*	0.047
DWI_reduced_ ADC	5.3 ± 2.2	21.9 ± 12.0	< 0.001	10.5 ± 5.5	34.1 ± 13.3	< 0.001	0.002	0.024
Kiwifruits, outer pericarp, site 2	ERC, *n* = 3 × 2 ROIs			noERC, *n* = 3 × 2 ROIs			ERC versus noERC	
	SNR, anterior (mean ± SD)	SNR, posterior (mean ± SD)	*p* value	SNR, anterior (mean ± SD)	SNR, posterior (mean ± SD	*p* value	SNR, anterior*, p* value	SNR, posterior*, p* value
T2WI	19.8 ± 5.1	17.4 ± 2.9	*0.485*	14.5 ± 6.8	18.5 ± 6.7	*0.255*	*0.481*	*0.691*
DWI_reduced_ *b* = 50	8.8 ± 1.5	7.1 ± 1.4	*0.421*	6.6 ± 3.1	8.4 ± 1.5	*0.173*	*0.463*	*0.178*
DWI_reduced_ *b* = 800	5.3 ± 4.2	8.2 ± 1.2	*0.401*	9.9 ± 0.5	7.8 ± 1.4	*0.076*	*0.225*	*0.708*
DWI_reduced_ ADC	10.8 ± 3.0	41.7 ± 13.1	*0.060*	5.2 ± 0.6	26.7 ± 9.8	*0.071*	*0.095*	*0.366*
Kiwifruits, peripheral placenta, site 1	ERC, *n* = 12 × 2 ROIs			noERC, *n* = 12 × 2 ROIs			ERC versus noERC	
	SNR, anterior (mean ± SD)	SNR, posterior (mean ± SD)	*p* value	SNR, anterior (mean ± SD)	SNR, posterior (mean ± SD	*p* value	SNR, anterior, *p* value	SNR, posterior*, p* value
T2WI	9.4 ± 3.9	8.6 ± 2.6	*0.251*	7.8 ± 2.6	8.1 ± 2.9	*0.715*	*0.285*	*0.496*
DWI_reduced_ *b* = 50	7.6 ± 4.3	6.1 ± 1.9	*0.332*	9.1 ± 2.8	9.1 ± 4.9	*0.992*	*0.379*	*0.066*
DWI_reduced_ *b* = 800	7.8 ± 3.1	7.0 ± 3.1	*0.581*	10.5 ± 2.9	11.5 ± 6.2	*0.529*	0.046	*0.086*
DWI_reduced_ ADC	15.1 ± 8.2	15.2 ± 8.6	*0.941*	26.7 ± 16.1	25.5 ± 9.2	*0.810*	0.036	0.010
Kiwifruits, peripheral placenta, site 2	ERC, *n* = 3 × 2 ROIs			noERC, *n* = 3 × 2 ROIs			ERC versus noERC	
	SNR, anterior (mean ± SD)	SNR, posterior (mean ± SD)	*p* value	SNR, anterior (mean ± SD)	SNR, posterior (mean ± SD)	*p* value	SNR, anterior*, p* value	SNR, posterior, *p* value
T2WI	9.0 ± 0.7	9.8 ± 3.5	*0.713*	9.0 ± 0.7	10.4 ± 2.9	*0.391*	*0.972*	*0.225*
DWI_reduced_ *b* = 50	7.1 ± 2.0	6.8 ± 0.2	*0.825*	6.5 ± 1.8	8.7 ± 2.1	0.010	*0.125*	*0.241*
DWI_reduced_ *b* = 800	7.2 ± 1.3	6.9 ± 2.2	*0.861*	8.6 ± 0.4	8.4 ± 1.6	*0.806*	*0.183*	*0.450*
DWI_reduced_ ADC	24.7 ± 2.2	35.6 ± 14.0	*0.277*	22.5 ± 11.4	22.1 ± 4.3	*0.937*	*0.729*	*0.251*

*p* values were obtained using Student’s *t* test for paired samples

*ADC* apparent diffusion coefficient, *DWI*_*reduced*_ echo-planar diffusion-weighted imaging with reduced field of view and selective excitation, *ERC* with endorectal coil, *noERC* without endorectal coil, *ROI* region-of-interest, *SD* standard deviation, *SNR* signal-to-noise ratio, *T2WI* T2-weighted turbo-spin-echo imaging

**Table 3:** Signal-to-noise ratio in human prostates with benign hyperplasia examined with endorectal coil filled with air in site 2 (*n* = 66 corresponding regions of interests, in anterior and posterior locations, respectively), and without endorectal coil at site 1 (*n* = 60 corresponding regions of interest, in anterior and posterior locations, respectively)

Prostates, peripheral zone	ERC, site 2, *n* = 66 × 2			noERC, site 1, *n* = 60 × 2		
	SNR, PZa (mean ± SD)	SNR, PZpl (mean ± SD)	*p* value	SNR, Pza (mean ± SD)	SNR, PZpl (mean ± SD)	*p* value
T2WI	6.2 ± 2.6	7.0 ± 3.0	*0.105*	4.4 ± 1.2	6.0 ± 2.8	< 0.001
DWI_reduced_ *b* = 50	4.9 ± 2.2	5.8 ± 3.0	0.026	5.8 ± 4.2	5.4 ± 2.7	*0.558*
DWI_reduced_ *b* = 800	8.0 ± 4.3	11.0 ± 6.0	0.001	12.6 ± 7.1	12.6 ± 6.5	*0.964*
DWI_reduced_ ADC	6.0 ± 2.3	8.2 ± 4.1	< 0.001	6.4 ± 4.4	9.3 ± 12.3	*0.096*
Prostates, transitional zone	ERC, site 2, *n* = 66 × 2			noERC, site 1, *n* = 60 × 2		
	SNR, Tza (mean ± SD)	SNR, TZp (mean ± SD)	*p* value	SNR, Tza (mean ± SD)	SNR, TZp (mean ± SD)	*p* value
T2WI	8.3 ± 3.5	7.6 ± 3.2	*0.155*	5.1 ± 1.9	6.1 ± 6.4	*0.218*
DWI_reduced_ *b* = 50	7.5 ± 2.8	7.1 ± 2.7	*0.292*	8.4 ± 3.7	8.6 ± 3.9	*0.688*
DWI_reduced_ *b* = 800	13.5 ± 5.4	13.7 ± 5.0	*0.796*	16.5 ± 5.8	16.5 ± 6.1	*0.972*
DWI_reduced_ ADC	9.4 ± 3.5	8.7 ± 3.6	*0.271*	9.2 ± 3.5	11.1 ± 9.8	*0.121*

*p* values were obtained using Student’s *t* test for paired samples

*ADC* apparent diffusion coefficient, *DWI*_*reduced*_ echo-planar diffusion-weighted imaging with reduced field of view and selective excitation, *ERC* with endo-rectal coil, *noERC* without endo-rectal coil, *Pza* anterior region of peripheral zone of human prostate, *PZpl* posterior-lateral region of peripheral zone of human prostate, *SNR* signal-to-noise ratio, *T2WI* T2-weighted turbo-spin-echo imaging, *Tza* anterior region of transitional zone of human prostate; *TZp* posterior region of transitional zone of human prostate

**Table 4:** Signal-to-noise ratios in one kiwifruit examined with an endorectal coil filled with air at both site 1 and site 2, and with perfluorocarbon in site 2

Kiwifruit, outer pericarp (*n* = 3 × 2 ROIs)	With air-filled ERC, site 1			With air-filled ERC, site 2			With PFC-filled ERC, site 2		
	SNR, anterior (mean ± SD)	SNR, posterior (mean ± SD)	*p* value	SNR, anterior (mean ± SD)	SNR, posterior (mean ± SD)	*p* value	SNR, anterior (mean ± SD)	SNR, posterior (mean ± SD)	*p* value
T2WI	16.0 ± 1.3	16.7 ± 2.2	*0.605*	15.4 ± 2.9	9.0 ± 1.3	0.047	12.2 ± 1.7	11.8 ± 0.4	*0.655*
DWI_reduced_ b = 50	4.0 ± 1.2	7.0 ± 2.1	*0.153*	6.2 ± 3.3	8.3 ± 1.6	*0.401*	8.2 ± 1.5	9.3 ± 3.0	*0.339*
DWI_reduced_ b = 800	4.6 ± 3.4	6.0 ± 1.0	*0.625*	9.9 ± 2.4	9.5 ± 2.3	*0.596*	9.7 ± 2.4	8.0 ± 1.6	*0.085*
DWI_reduced_ ADC	7.2 ± 1.3	29.5 ± 12.9	*0.113*	12.9 ± 2.9	40.3 ± 22.5	*0.203*	13.6 ± 6.2	56.8 ± 9.1	0.040
Kiwifruit, outer pericarp	ERC air site 1 versus site 2					ERC air versus PFC site 2			
	SNR, anterior*, p* value		SNR, posterior, *p* value			SNR, anterior, *p* value		SNR, posterior, *p* value	
T2WI	*0.600*		**0.007**			*0.347*		*0.091*	
DWI_reduced_ *b* = 50	*0.502*		*0.577*			*0.446*		*0.759*	
DWI_reduced_ *b* = 800	*0.011*		*0.214*			*0.841*		*0.077*	
DWI_reduced_ ADC	*0.109*		*0.563*			*0.849*		*0.260*	
Kiwifruit, peripheral placenta (*n* = 3 × 2 ROIs)	With air-filled ERC, site 2			With air-filled ERC, site 2			With PFC-filled ERC, site 2		
	SNR, anterior (mean ± SD)	SNR, posterior (mean ± SD)	*p* value	SNR, anterior (mean ± SD)	SNR, posterior (mean ± SD)	*p* value	SNR, anterior (mean ± SD)	SNR, posterior (mean ± SD)	*p* value
T2WI	9.8 ± 2.8	9.7 ± 3.3	*0.831*	7.5 ± 0.7	8.7 ± 0.7	*0.115*	6.0 ± 0.6	7.0 ± 2.0	*0.370*
DWI_reduced_ *b* = 50	5.9 ± 2.0	6.0 ± 0.6	*0.970*	9.2 ± 1.1	7.4 ± 1.4	*0.163*	8.9 ± 1.3	5.9 ± 0.8	*0.069*
DWI_reduced_ *b* = 800	7.0 ± 0.9	5.3 ± 1.4	*0.247*	9.9 ± 0.9	8.0 ± 2.8	*0.238*	11.8 ± 2.2	6.8 ± 1.2	*0.070*
DWI_reduced_ ADC	22.5 ± 8.4	21.8 ± 7.5	*0.853*	28.5 ± 12.3	23.2 ± 9.7	*0.718*	25.0 ± 13.0	25.9 ± 3.9	*0.882*
Kiwifruit, peripheral placenta	ERC air site 1 versus site 2					ERC air versus PFC site 2			
	SNR, anterior, *p* value		SNR, posterior, *p* value			SNR, anterior, *p* value	SNR, posterior*, p* value		
T2WI	*0.324*		*0.565*			*0.148*	*0.378*		
DWI_reduced_ *b* = 50	*0.211*		*0.297*			*0.825*	*0.336*		
DWI_reduced_ *b* = 800	*0.108*		*0.230*			*0.257*	*0.406*		
DWI_reduced_ ADC	*0.377*		*0.851*			*0.155*	*0.763*		

*p* values were obtained using Student’s *t* test for paired samples

*ADC* apparent diffusion coefficient, *DWI*_*reduced*_ echo-planar diffusion-weighted imaging with reduced field of view and selective excitation, *ERC* endorectal coil, *PFC* perfluorocarbon, *ROI* region of interest, *SD* standard deviation, *SNR* signal-to-noise ratio, *T2WI* T2-weighted turbo-spin-echo imaging

**Table 5:** Diameters and volumes of kiwifruits (*n* = 5) and human prostates (without endorectal coil, *n* = 10; with endorectal coil, *n* = 11)

	Diameter (mean ± SD)			Volume (mean ± SD) (cm^3^)
	AP (cm)	LR (cm)	CC (cm)	
Kiwifruits, noERC	4.9 ± 0.1	5.3 ± 0.2	6.5 ± 0.5	89.2 ± 11.2
Kiwifruits,_ERC	4.8 ± 0.1	5.4 ± 0.2	6.5 ± 0.4	89.7 ± 9.5
Prostates,_noERC	4.6 ± 1.0	6.0 ± 1.1	5.7 ± 1.3	90.8 ± 48.5
Prostates, ERC	4.2 ± 0.9	6.0 ± 0.5	6.5 ± 1.0	87.5 ± 35.7
	*p* values			
Kiwifruits_noERC/ERC	*0.178*	*0.142*	*0.621*	*0.638*
Prostates,_noERC/ERC	*0.270*	*0.902*	*0.146*	*0.863*
noERC, kiwifruits versus prostates	*0.430*	*0.080*	*0.086*	*0.920*
ERC, kiwifruits versus prostates	0.028	0.005	*0.860*	*0.853*

*p* values were obtained using Student’s *t* test

*AP* anterior-posterior, *CC* cranio-caudal, *ERC* with endorectal coil, *LR* left-to-right, *noERC* without endorectal coil, *SD* standard deviation

## Results

### Main findings

**Fig. 3:**
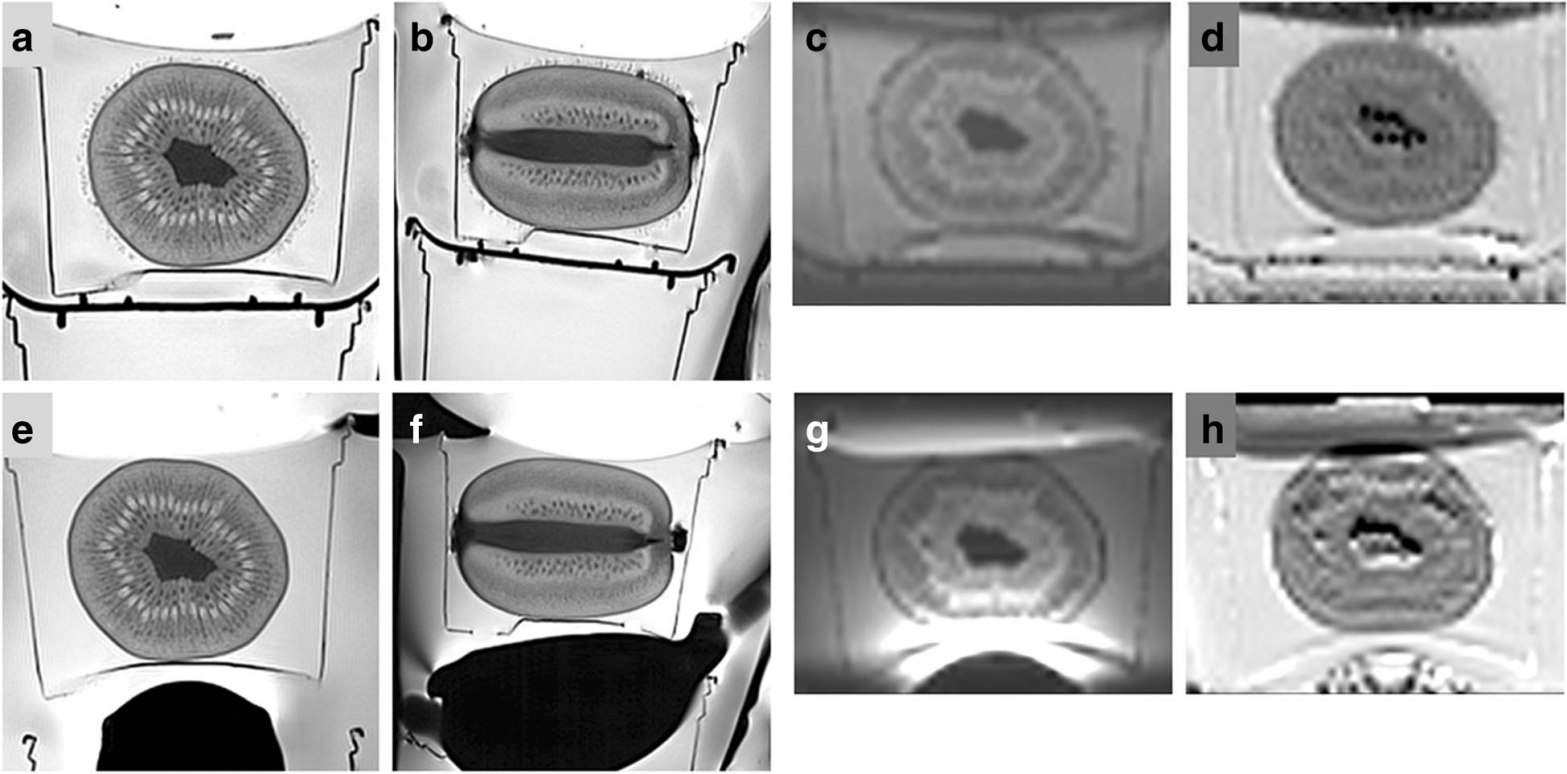
Biparametric magnetic resonance imaging at 3.0 T of a green Hayward kiwifruit (*Actinidia deliciosa*) was performed at site 1 both without (**a**–**d**) and with an endo-rectal coil (**e**–**h**) included in a multichannel phased-array coil system: axial (**a**, **e**) and sagittal (**b**, **f**) T2-weighted imaging (T2WI), axial diffusion-weighted imaging with a *b* value of 800 s/mm^2^ (**c**, **g**), and corresponding apparent diffusion coefficient map (**d**, **h**) obtained with a reduced field of view

**Fig. 4:**
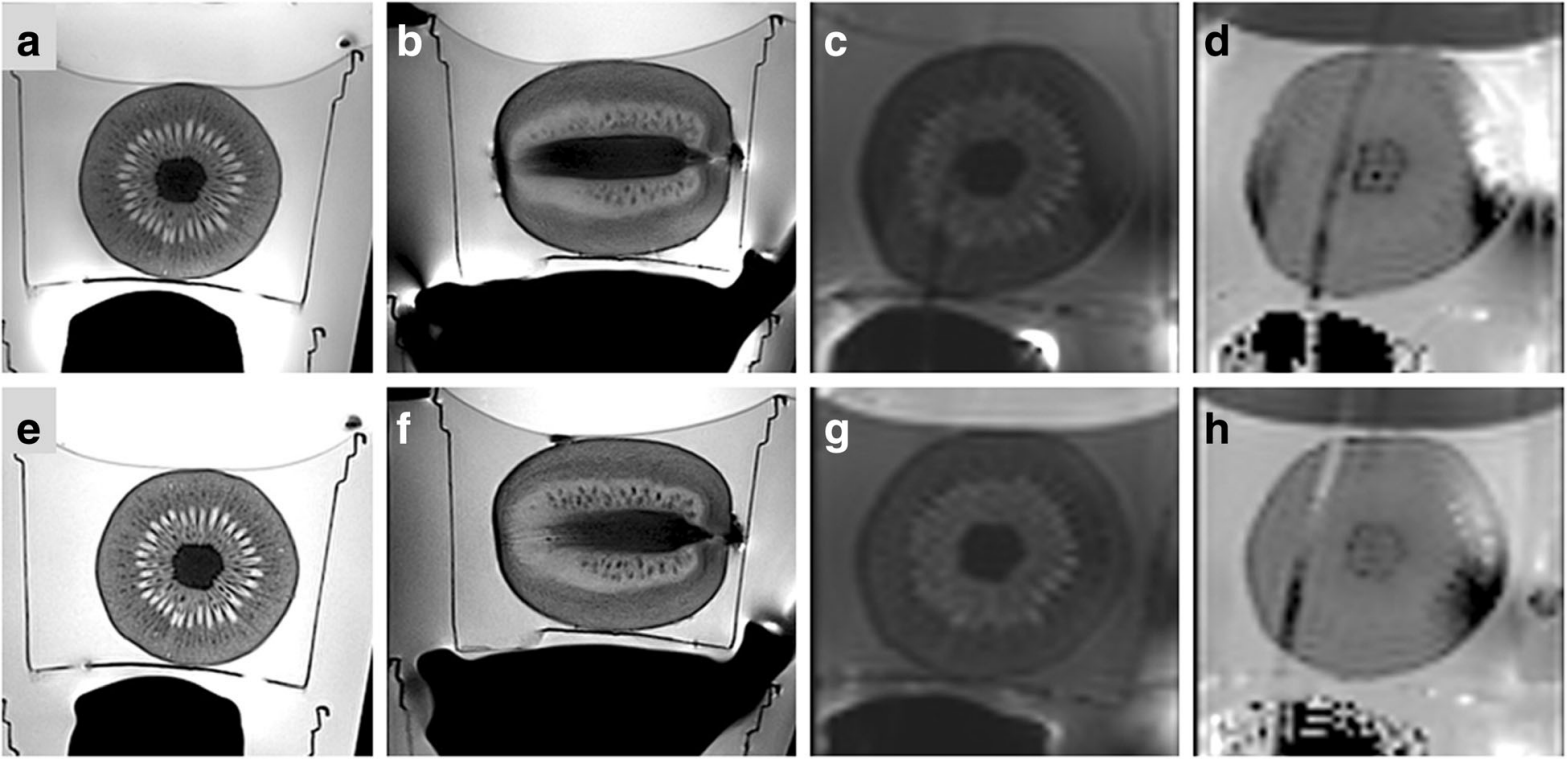
Biparametric magnetic resonance imaging at 3.0 T in a green Hayward kiwifruit (*Actinidia deliciosa*) was performed at site 2 using an endorectal coil included in a multichannel phased-array coil system. Images in the upper panel (**a**–**d**) were obtained with air inside the endorectal coil balloon, while images in the lower panel (**e**–**h**) were obtained with perfluorocarbon inside the endorectal-coil balloon: axial (**a**, **e**) and sagittal (**b**, **f**) T2-weighted imaging, axial diffusion-weighted imaging with a *b* value of 800 s/mm^2^ (**c**, **g** ), and corresponding apparent diffusion coefficient map (**d**, **h**) obtained with a reduced field of view

**Fig. 5:**
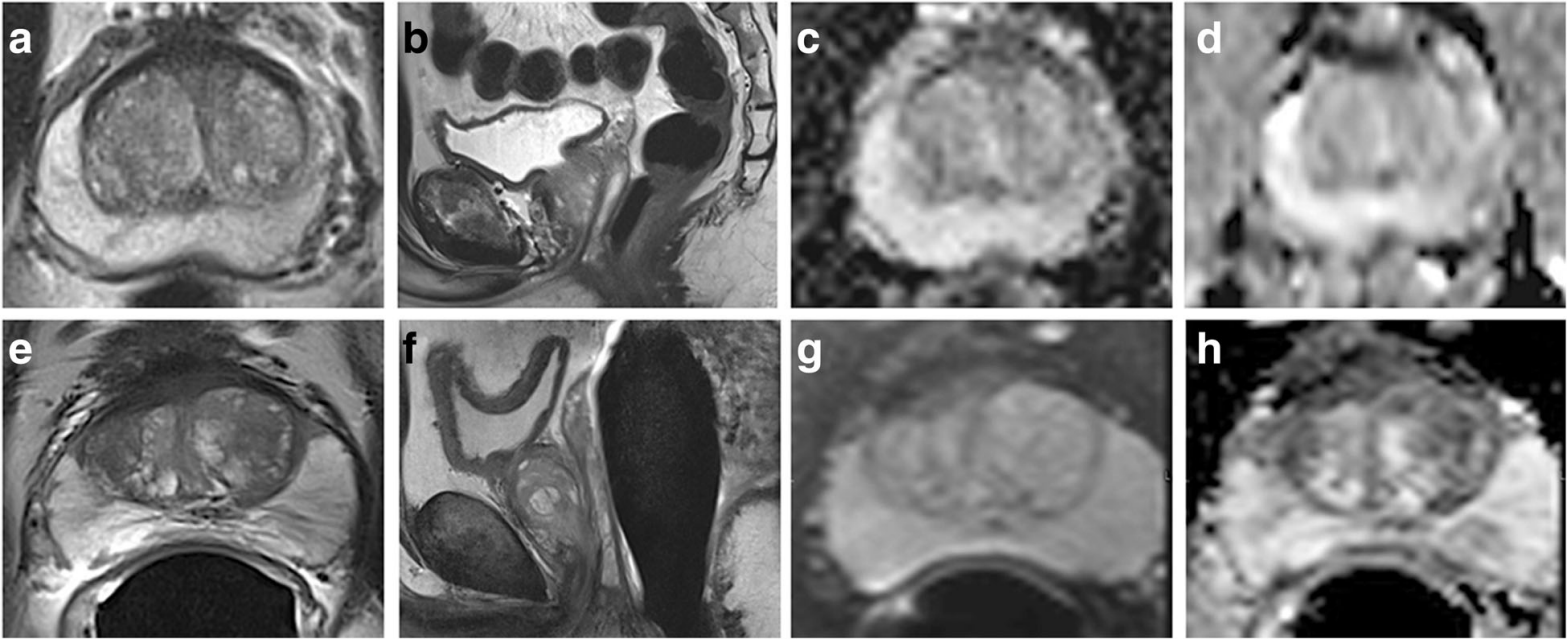
Biparametric magnetic resonance imaging at 3.0 T in patients with benign prostate hyperplasia was performed without endorectal coil at site 1 (**a**–**d**) and with endorectal coil at site 2 (**e**–**h**) included in a multichannel phased-array coil system: axial (**a**, **e**) and sagittal (**b**, **f**) T2-weighted imaging, axial diffusion-weighted imaging with a *b* value of 800 s/mm^2^ (**c**, **g**), and corresponding apparent diffusion coefficient map (**d**, **h**) obtained with a reduced field of view

Results obtained in kiwifruits and human prostates are displayed in Tables 2 and 3, and in Figs. 3, 4, 5, respectively. In both T2WI and DWI_reduced_, with low and high *b* values and ADC maps, respectively, SNR levels were roughly similar in kiwifruits and human prostates.

### T2-weighted imaging

Both with and without ERC, T2WI in kiwifruits showed similar SNR in the anterior and posterior OP and PP at both sites (see Table 2). T2WI findings in TZ of prostates corroborated results in PP of kiwifruits at both sites. However, SNR was significantly higher in the posterior than anterior PZ of prostates examined without ERC, unlike in OP of kiwifruits (Table 3).

### Diffusion-weighted imaging

In DWI_reduced_ with low *b* value, ERC application showed no significant increase in SNR in kiwifruits. Kiwifruits demonstrated a significantly higher SNR in the anterior OP without than with ERC at site 1, but not at site 2. There was not significantly higher SNR in the posterior versus anterior OP with ERC at site 1, and in the posterior versus anterior PP without ERC at site 2. There were no other differences (Table 2). Similarly, prostates showed not significantly higher SNR in the posterior than anterior PZ with ERC, but not without ERC, and no significant differences in the TZ (Table 3).

In DWI_reduced_ with high *b* value, ERC application showed no significant increase in SNR in kiwifruits. Kiwifruits demonstrated not significantly higher SNR in the posterior than anterior OP without ERC, the posterior OP without versus with ERC, and the anterior PP without versus with ERC at site 1 (Table 2). There were no other differences. In prostates, however, there was significantly higher SNR in the posterior versus anterior PZ with ERC, while there were no differences without ERC (Table 3).

Both with and without ERC, ADC maps of kiwifruits showed higher SNR in the posterior than anterior OP. Differences were statistically significant at site 1. SNR in ADC maps was higher without than with ERC at site 1. There were no other differences (Table 2). ADC maps of prostates showed higher SNR in posterior versus anterior PZ with ERC, but not without ERC. There were no other differences in PZ and TZ (Table 3).

### Endo-rectal coil filled with air versus perfluorocarbon in kiwifruit

One kiwifruit was examined with an air-filled ERC at both site 1 and site 2 and additionally with a perfluorocarbon-filled ERC at site 2. Except for a not significantly higher SNR in the anterior OP with an air-filled ERC at site 2 when compared to site 1, there were no differences in SNR (Table 4, Fig. 3).

### Potential confounders

Cranio-caudal diameters and volumes of kiwifruits and human prostates did not differ significantly with and without ERC. There were no significant differences in anterior-posterior and lateral diameters between kiwifruits and prostates examined without ERC. However, in kiwifruits and human prostates examined with ERC, lateral diameters were significantly larger in prostates, and anterior-posterior diameters were not significantly larger in kiwifruits (Table 5).

Lateral diameter of patients’ pelvis exceeded localiser dimensions in several cases examined with ERC at site 2, such that it was not possible to calculate mean ± SD. Pelvic anterior-posterior diameters were markedly but not significantly larger in patients examined without ERC. There were no significant differences between phantom diameters in kiwifruits examined with and without ERC and between patients’ pelvic diameters and kiwifruit phantom diameters in examinations without ERC. However, distance from the respective posterior surface of kiwifruits and prostates to the table top of the scanner table was significantly longer in kiwifruits with than without ERC and in patients versus kiwifruit phantoms examined with ERC; it was markedly, but not significantly, longer in patients versus kiwifruit phantoms examined without ERC (Table 6, Fig. 1).

**Table 6:** Diameters of kiwifruit phantom and human pelvis, and respective distances from the posterior surface of kiwifruit and human prostates to the scanner table

	Diameter (mean ± SD)		Distance from kiwifruit or prostate to scanner table (mean ± SD) (cm)
	AP (cm)	LR (cm)	
Kiwifruits,_noERC	21.6 ± 2.3	37.5 ± 2.3	8.0 ± 0.3
Kiwifruits, ERC	23.1 ± 2.4	36.9 ± 1.9	9.1 ± 0.2
Patients’_pelvis,_noERC	23.0 ± 2.4	39.7 ± 2.8	9.9 ± 0.9
Patients’_pelvis,_ERC	20.8 ± 1.4	>36.0	10.0 ± 0.8
	*p* values		
Kiwifruits,_noERC versus ERC	*0.184*	*0.430*	0.002
Patients, noERC versus ERC	0.021	n/a	*0.792*
noERC, kiwifruits versus patients	*0.102*	*0.417*	0.006
ERC, kiwifruits versus patients	*0.288*	n/a	< 0.001

*p* values were obtained using Student’s *t* test

*AP* anterior-posterior, *CC* cranio-caudal, *ERC* with endorectal coil, *LR* left-to-right, *noERC* without endorectal coil, *SD* standard deviation

## Discussion

The most important finding of this study was that overall, with minor exceptions, similar SNR was achieved with and without ERC in a biparametric protocol for MRI of the prostate at 3.0 T that included T2WI and DWI_reduced_ with low (50 s/mm^2^) and high (800 s/mm^2^) *b* values and monoexponential ADC maps, in a phantom including kiwifruits as substitutes for human prostates that was examined at two different centres. SNR levels were roughly similar in kiwifruits and human prostates in all sequences at both sites. With few exceptions, findings in kiwifruits were corroborated by retrospective analysis of SNR in human prostates examined with the same protocols at both sites.

This study has limitations. First, although measurements performed on the same model of 3.0-T systems with identical coil systems, albeit with small variations in sequence parameters, yielded similar results at two centres, it remains to be shown that kiwifruit phantoms generate comparable results on other MRI systems and with other magnetic field strengths, too. Second, the study was restricted to T2WI and DWI of the prostate and left out dynamic contrast-enhanced and spectroscopic sequences. However, both current guidelines and recent research emphasise the eminent role of T2WI and DWI in prostate imaging [4–7]. Third, the study included only echo-planar DWI sequences with selective excitation and reduced field of view. Therefore, while optimised DWI sequences were applied, neither standard DWI with a full field of view nor diffusion tensor imaging or non-echo-planar DWI was tested in this study [1, 4, 21–23]. Fourth, while it has been shown that human prostates *in vivo* are significantly compressed in anterior-posterior direction between ERC and symphysis pubis and decrease in volume [24], no change in diameters or volume occurred in kiwifruits. However, although anterior-posterior diameters were markedly shorter and lateral diameters significantly longer in prostates than in kiwifruits examined with ERC, their cranio-caudal diameters and volumes did not differ. Also, posterior-anterior gradients of SNR occurred in some sequences in both kiwifruits and human prostates and both with and without ERC, such that the compression of human prostates by ERC is an unlikely reason for this phenomenon. In support of this impression, it has previously been shown that the compression of the normal peripheral zone of the human prostate by an enlarged transitional zone does not alter T2 relaxation or ADC and therefore does not alter tumour-to-PZ contrast [25]. Fifth, axial cross-sectional anatomy is concentric in kiwifruits but eccentric in human prostates. However, both feature zonal anatomy, with the OP and PP of kiwifruits resembling the PZ and TZ of human prostates, respectively [14, 26, 27]. Also, SNR levels were roughly similar in kiwifruits and human prostates in all sequences at both sites. Sixth, the respective surroundings of kiwifruits in the phantom and prostates in the human pelvis differed. Therefore, the kiwifruit phantom as applied here may not appear suitable to imitate human tissue surrounding the prostate. However, anterior-posterior and lateral dimensions of the kiwifruit phantom were like respective human pelvic dimensions, such that the coil load was similar in this study.

Seventh, longer distance from the scanner table to the posterior surface of prostates when compared with kiwifruits affected examinations with and without ERC. However, since SNR levels were roughly similar in kiwifruits and human prostates in all sequences at both sites, with and without ERC, no specific confounding effect could be derived. Eighth, SNR in tissue, with noise defined as being the first SD of SI, is affected by both random noise, as an overall measure of technical image quality, and biological tissue homogeneity. However, this lack of separation affected both kiwifruits and human prostates similarly under all conditions tested here. Also, since kiwifruits were individually examined both with and without ERC within the same phantom, and respective SNR levels were intra-individually compared for ROIs in the same locations and subjected to statistical tests for paired samples, the influence of biological tissue homogeneity on intra-individual SNR change should be reasonably reduced, and the influence of change in technical image quality should be emphasised. Again, since SNR levels were roughly similar in kiwifruits and human prostates in all sequences in both sites, with and without ERC, no specific confounding effect could be derived. Ninth, this study included a higher number of human prostates than kiwifruits. Also, prostates were selected for size and lack of focal pathology to increase the chance of having a homogenous sample. These measures were taken to account for the higher likelihood of biological variability among human prostates [15–17] than among kiwifruits [18]. Tenth, examinations in this study were consequently limited to large prostates with benign prostate hyperplasia and kiwifruits of similar volume. Therefore, it cannot be ruled out that results would differ in much smaller or much larger kiwifruits and prostates.

Within these limitations, this experimental study in kiwifruits suggests that MRI examinations at 3.0 T with a biparametric imaging protocol involving T2WI and DWI_reduced_ yield technically similar results both with and without ERC included in a multichannel phased-array coil system. Due to the restricted biological variability of commercially available kiwifruits [18] and the option to standardise setup of the kiwifruit phantom for prostate imaging [14], comparative tests of MRI technology should emphasise technical rather than biological differences when kiwifruits are examined instead of human prostates. Retrospective comparison of findings with selected human prostates with benign prostate hyperplasia corroborated experimental study results in kiwifruits.

ERC application in 3.0-T prostate MRI is one area of ongoing debate [3, 28]. One recent survey among 128 institutions reports that 36 (28%) examined prostates at 3.0 T, but only two (1.5% of all) applied ERCs at 3.0 T [2]. Another survey among 107 institutions found that 5.8% examined prostates with ERC at 3.0 T and 63.5% without ERC at 3.0 T [8]. While some authors advocate ERC application due to better test quality parameters [9–11], others found no improvement over MRI without ERC [5]. Although some researchers report better subjective image quality ratings with ERC [5], others found no objective SNR advantages [29]. Our study by design cannot generate test-quality parameters for prostate MRI. However, our preliminary data in both kiwifruits and prostates with benign prostate hyperplasia support the view that there is no SNR advantage in using an ERC at 3.0 T, at least with the types of scanner, coil system, and T2WI and DWI sequences applied here. Thus, unless technical reasons other than SNR can be identified that would explain why ERC application should improve test quality parameters for prostate MRI at 3.0 T, it must be assumed that differences in prostate MRI with and without an ERC reported by some researchers [9–11] but not corroborated by others [5] may be subjective, or due to training and habituation.

Findings in one kiwifruit of similar SNR in T2WI, DWI_reduced_, and ADC maps with perfluorocarbon when compared with air in the ERC balloon differ from a previous report of decrease in posterior-anterior field gradient and significant improvement in field homogeneity with barium suspension or perfluorocarbon relative to air in the ERC balloon [30], although no sound explanation can currently be offered.

In conclusion, our findings in both kiwifruits and prostates with benign prostate hyperplasia suggest that MRI examinations at 3.0 T with a biparametric imaging protocol, including T2WI and DWI_reduced_, yield technically equivalent results both with and without ERC. The added value of applying commercially available kiwifruits, with their limited biological variability that is governed by pertinent European Union regulation, as experimental phantom substitutes for human prostates lies in the emphasis on technical aspects due to reduction of confounding that derives from biological variability in human prostates. Results imply that DWI_reduced_ of the prostate at 3.0 T without ERC may be advantageous even in a clinical setting because it may increase SNR in ADC maps and provide similar posterior and better anterior SNR when compared to MRI with ERC. However, studies of MRI protocols for prostate imaging involving kiwifruits as substitutes for human prostates should be extended to other MRI systems and additional sequences for validation.

## Abbreviations

ADC: Apparent diffusion coefficient
DWI: Diffusion-weighted imaging
DWI_reduced_: DWI with reduced field-of-view
mpMRI: Multi-parametric MRI
MRI: Magnetic resonance imaging
PI-RADS-v2: Prostate imaging reporting and data system, version 2
PZ: Peripheral zone
ROI: Region of interest
SD: Standard deviation
SI: Signal intensity
SNR: Signal-to-noise ratio
T2WI: T2-weighted imaging
TZ: Transitional zone
